# Wholesale Prophylactic Treatment of Miasmatic Diseases by the Removal of the Cause—Completely Successful Result

**Published:** 1860-10

**Authors:** J. T. Calhoun

**Affiliations:** Rahway, N. J.


*Wholesale Prophylactic Treatment of Miasmatic Diseases by the Removal of the Cause—Completely Successful Result.* By J. T. CALHOUN, M. D., of Rahway, N. J.

It is the popular opinion, that the mission of the physician is merely to heal the sick. We of the profession know that it extends beyond this narrow sphere, and that it is as much our duty to prevent disease as it is to treat it when beyond prevention. The prophylactic treatment is much the more important, and it is with no little satisfaction, as a medical man, that I at this time record, in the annals of the profession, one of the most complete and successful attempts to prevent disease, on record, if, indeed, there is recorded any case where the attempt to prevent miasmatic diseases over a number of square miles of country has been crowned with such complete and positive success.

The city of Rahway, at this time containing some seven thousand inhabitants, is one of the suburban cities which dot the northern part of New Jersey within a radius of from 20 to 40 miles from the great Empire city, and which are, in reality, but suburbs of that great commercial centre. It has but recently donned a corporate garb; but, as a village, it has long been noted for two things: the manufacture of carriages, and miasmatic diseases. Through its centre runs a little stream, dignified with the name of river, which, with its branches, was crossed by five dams. These dams flooded many acres of ground, and from these partially flooded meadows was generated the miasmatic poison. The reputation of the village for miasmatic diseases, was well deserved. A stranger entering it but for a few days even, was sure to be attacked with one form or other of malarious disease. The old settlers, (those who were acclimated,) as a general rule, were comparatively free from such diseases. We say comparatively exempt only when considering the liability of new comers to be attacked, for it was no uncommon thing for whole families, residents for years, to be prostrate with intermittent or remittent fever. A person moving into the village, was *sure* to be attacked ere a year had passed over his head. The autumnal season was certain to affect him, even if he had passed through the spring and summer unscathed. It does not take long for a bad name to spread, and when as well deserved as it was in this case, it was no wonder that, for miles around, the very name of Rahway was synonymous with sickness. No person walking through the village, on an autumn evening, could fail to discern the cause for this pitiable condition of health. The cause was obvious to all his senses. He could *see* the fog rising from the surface of the ponds, and rolling along with the gentle breeze; he could *feel* the moisture as it rolled over him, and he inhaled with every breath its poison-loaded contents, and he could *smell* the odor of decaying vegetation.

It was a fact patent to every one, that the cause must be removed ere the sickness was got rid of, and be got rid of it must, for, while a paradise for doctors, the place was a pest-house for the people. The physicians did not hesitate in pointing out the remedy, which was to remove the dams and drain the overflowed meadows. The advent of the deadly, and hitherto almost unknown, pernicious fever, added to the already too grievous intermittent and remittent, formed a burden too weighty to be borne; and, in the fall of 1853, a public meeting was called to take measures for the removal of the dams. The physicians to a man, to their praise be it spoken, strongly urged the measure, although they knew it would reduce their income. The movement was strongly opposed. To the physicians improper motives were imputed, and every means was used to thwart the designs of the friends of the proposed removal. The movement, however, went steadily onward. Dr. David S. Craig, a retired physician, introduced a bill into the Legislature, of which body he was at that time a member, granting authority to remove the dams, and by his aid, backed by many public-spirited citizens, the bill was passed. From some legal reason, it proved to be inoperative, and a supplement was passed the following winter to make the act operative. Its constitutionality was then contested; but, on the 1st of February, 1855, the exertions of the friends of the measure were brought to a successful result by the removal of two of the dams. They had triumphed over every obstacle.

The following winter two more of the dams were removed—the whole at a cost of between $30,000 and $35,000. The fifth dam, from its location and surroundings, was deemed but of little harm, and was allowed to remain. Many were the gloomy forebodings as to the result—many the evil prognostications by the unbelievers; but the succeeding summer and autumn rolled around but to dissipate all fears, and to establish the fact that the removal of the dams was right, and that the result more than fulfilled the promises of health which had been held out. Rahway changed from one of the sickliest to one of the most healthful towns in this State.

Sufficient time has now elapsed to show that the good results were not temporary. Formerly, chills and fever was a household friend, if I may so express it. Every family knew enough to diagnose them without difficulty. The Irish servant girls, as a class, were probably oftener affected than any other class of persons. Remittent fever was, next to intermittent, the most common disease the physician was called upon to treat. All diseases were, more or less, altered in their nature or affection in some way by this miasmatic poison. Such *was* the state of things. Mark the contrast!—*Now* a case of miasmatic disease is rarely seen. A few sporadic cases now and then occur; but they are few and far between. "The chills" are unknown in the family. The doctors, from being "driven to death," are now enabled "to take their time." What has been the exact decrease, I am unable to determine. I have heard it said by one physician, that we have not one case of miasmatic disease now where there were formerly a thousand. I believe myself, that, if we place it at one case now where formerly were 500, we should not be very far from the mark. This may appear extravagant to a stranger; but, when I look back and think of the vast multitude of cases never seen by physicians, but treated by domestic remedies or quack medicines, I feel convinced that such an estimate is not far from correct. To mention but a single fact as corroborative proof, Mr. A. C. Watson, the principal druggist, tells me that, before the removal of the dams, he sold, on an average, one ounce of quinine a day, to say nothing of the pounds of bark and its preparations, with countless quantities of cholagogues, and the hundred other empirical tonic mixtures and pills. Now he sells an ounce of quinine about *every three months*, and scarcely ever has a call for any of the empirical anti-periodical preparations. But, assuming even, that only one case of miasmatic disease occurs now where a hundred were formerly seen, (for I do not wish to exaggerate, even unintentionally, and it is better to be within than beyond the mark,) and how great and incalculable has been the benefit! The physicians and druggists alone have suffered; the benefit to the citizens has been incalculable—where once were acres of decaying vegetation, generating the pestilential malaria, now are seen fields of waving grain; and on the spots over which in my boyhood I have skated, now are seen large factories, resounding with the ring of the anvil or the stroke of the hammer. I have no doubt that the people of Rahway have already been more than repaid for the outlay incident to this improvement—to say nothing of the rise of property, the saving to the mechanic of the many days annually lost by sickness, will more than pay it. It is a strange fact, that there has been very little sickness of any kind since the removal of the dams—whether attributable to that cause or not, may perhaps be questionable. Epidemics of scarlatina and variola have visited neighboring cities and villages, and we have been exempt. We have become emphatically a healthy city.

I cannot close this article without an humble tribute to two veteran members of our profession, who stood forth prominently among the strong advocates of this important measure. It may, perhaps, be safely stated, that, without the help of Drs. Moses Jacques and David S. Craig, this work would not have been completed, and our village would have remained the same pestilential hole it was for so long a time. Aged physicians, both of them, retired from the active duties of their profession, they returned again to the contest with disease, and obtained over it a victory before which their former triumphs were as nothing, and which will tell on all future generations, and remain a monument to their memories long after they have passed from the stage of life. One (Dr. Jaques) is already an inhabitant of the silent city of the dead. Dr. Craig still lives to witness the complete success of his efforts, and, although prevented by an intracapsular fracture of the femur from taking an active part in every good work, as was his wont, he is still spared to witness some of the blessings he did so much to confer upon all future generations.—*Med. & Surg. Reporter.*

